# Biomarkers of neurodegeneration and glial activation validated in Alzheimer’s disease assessed in longitudinal cerebrospinal fluid samples of Parkinson’s disease

**DOI:** 10.1371/journal.pone.0257372

**Published:** 2021-10-07

**Authors:** Michael Bartl, Mohammed Dakna, Douglas Galasko, Samantha J. Hutten, Tatiana Foroud, Marian Quan, Kenneth Marek, Andrew Siderowf, Jonas Franz, Claudia Trenkwalder, Brit Mollenhauer, on behalf of the Parkinson’s Progression Markers Initiative

**Affiliations:** 1 Department of Neurology, University Medical Center Goettingen, Goettingen, Germany; 2 Department of Neurosciences, University of California, San Diego, San Diego, CA, United States of America; 3 The Michael J. Fox Foundation for Parkinson’s Research, New York, NY, United States of America; 4 Department of Medical and Molecular Genetics, Indiana University School of Medicine, Indianapolis, IN, United States of America; 5 Roche Diagnostics, Indianapolis, IN, United States of America; 6 Institute for Neurodegenerative Disorders, New Haven, CT, United States of America; 7 Department of Neurology, Perelman School of Medicine, University of Pennsylvania, Philadelphia, PA, United States of America; 8 Institute of Neuropathology, University Medical Center Göttingen, Göttingen, Germany; 9 Campus Institute for Dynamics of Biological Networks, University of Göttingen, Göttingen, Germany; 10 Max Planck Institute for Experimental Medicine, Göttingen, Germany; 11 Paracelsus-Elena-Klinik, Kassel, Germany; 12 Department of Neurosurgery, University Medical Center Goettingen, Goettingen, Germany; University of Rome Tor Vergata, ITALY

## Abstract

**Aim:**

Several pathophysiological processes are involved in Parkinson’s disease (PD) and could inform *in vivo* biomarkers. We assessed an established biomarker panel, validated in Alzheimer’s Disease, in a PD cohort.

**Methods:**

Longitudinal cerebrospinal fluid (CSF) samples from PPMI (252 PD, 115 healthy controls, HC) were analyzed at six timepoints (baseline, 6, 12, 24, 36, and 48 months follow-up) using Elecsys® electrochemiluminescence immunoassays to quantify neurofilament light chain (NfL), soluble TREM2 receptor (sTREM2), chitinase-3-like protein 1 (YKL40), glial fibrillary acidic protein (GFAP), interleukin-6 (IL-6), S100, and total α-synuclein (αSyn).

**Results:**

αSyn was significantly lower in PD (mean 103 pg/ml vs. HC: 127 pg/ml, p<0.01; area under the curve [AUC]: 0.64), while all other biomarkers were not significantly different (AUC NfL: 0.49, sTREM2: 0.54, YKL40: 0.57, GFAP: 0.55, IL-6: 0.53, S100: 0.54, p>0.05) and none showed a significant difference longitudinally. We found significantly higher levels of all these markers between PD patients who developed cognitive decline during follow-up, except for αSyn and IL-6.

**Conclusion:**

Except for αSyn, the additional biomarkers did not differentiate PD and HC, and none showed longitudinal differences, but most markers predict cognitive decline in PD during follow-up.

## Introduction

Although the etiology of Parkinson’s disease (PD) is not yet fully elucidated, evidence suggests that α-synuclein (αSyn) forms aggregates causing axonal and neuronal damage, leading to progressive neuronal loss and PD symptoms [1]. αSyn also shows a gene dosage effect [2] and therefore its levels in cerebrospinal fluid (CSF) have been extensively studied in several independent cohorts in the past, including the Parkinson Progression Marker Initiative (PPMI). Levels of αSyn are decreased in PD but overlap with healthy subjects and show minimal longitudinal changes [3, 4], highlighting the need for additional biomarkers. Other than αSyn, multiple pathophysiological processes are involved in neurodegeneration and have informative biomarker potential, as shown in Alzheimer’s disease (AD) [5].

There are common features of neurodegeneration in PD and AD [6]. Up to 80% of PD patients show an AD-like pathology with a prominent accumulation of β-amyloid plaques and tau-protein containing neurofibrillary tangles [7]. Additionally, a high percentage of patients with PD experience cognitive decline or even dementia during the disease course [8, 9]. The Roche NeuroToolKit (NTK) is a panel of automated robust prototype assays that quantifies several established biomarkers of axonal damage (neurofilament light [NfL]), microglial- (soluble triggering receptor expressed on myeloid cells 2 [sTREM2]) and astroglial-related response (glial fibrillary acidic protein [GFAP], chitinase-3-like protein 1 [YKL40], S100), neuroinflammation (interleukin 6 [IL-6]), and αSyn. NTK has been studied in AD, where an initial decrease in β-amyloid_1-42_ (a-beta) was followed by an increase of total-tau (t-tau) and phosphorylated-tau-181 (p-tau), and increases in synaptic biomarkers (Ng) and those reflecting neuroinflammation, which was pronounced in a-beta-positive individuals. Further, markers of neurodegeneration and glial activation were elevated in p-tau-181/ β-amyloid_1-42_ positive subjects with mild cognitive impairment (MCI)/dementia and neurodegenerative proteins increased with clinical severity and predicted a cognitive decline in this group [10–12].

We aimed to investigate NTK in a multicenter, longitudinal cohort of recently diagnosed PD patients and matched healthy controls (HC) to evaluate its potential to differentiate between the groups and to detect longitudinal changes.

## Material & methods

### Participants and characteristics

PPMI is an ongoing prospective, longitudinal, observational, international multicenter trial that aims to identify PD biomarkers [13]. Recently diagnosed, unmedicated PD subjects were enrolled according to baseline (BL) inclusion criteria: (1) a recent idiopathic PD diagnosis, (2) no PD treatment, (3) dopamine transporter (DaT) SPECT deficit, and (4) no indication of dementia. Details of the aims, methodology, and scope of the study have been published previously [14]. PPMI is listed in clinicaltrials.gov as NCT01141023. Ethical approval was obtained by the institutional review boards at each site, for the Paracelsus Elena Klinik Kassel, this was the Ethics Committee of the State Medical Association of Hessen, Germany.

The participants provided written informed consent.

The analyzed dataset was downloaded on 2/23/2020. Samples were selected randomly. Motor function was assessed with the Movement Disorders Society-Unified Parkinson’s Disease Rating Scale (MDS-UPDRS) part III and Total score [13]. Cognitive assessment included the Montreal Cognitive Assessment (MoCA) [15]. To detect a relevant cognitive impairment, we used a MoCA score < 26 as a cut-off value. This approach is based on the PD-MCI criteria published in 2012, corresponding to a Level I abbreviated assessment [16]. Further, we assessed the available data on genetic variations including Apolipoprotein E4 (ApoE4), GBA, LRRK2, SNCA, and R47H variant in the PD group.

Overall, four patients were retrospectively excluded from the analysis, three because of the later diagnosis of other neurological disorders.

### Biomarker measurements

Sample collection and processing were performed according to the PPMI biologics manual. Roche NTK is a panel of automated robust exploratory prototype sandwich immunoassays designed to evaluate biomarkers associated with key pathologic events characteristic of AD and other neurological disorders. It was used to quantify NfL, sTREM2, GFAP, IL-6, YKL40, S100, and αSyn. All measurements were performed in singlicate on a cobas e 411 analyzer at Covance Greenfield Laboratories (Translational Biomarker Solutions, Indiana, USA).

### Statistical analysis

Numerical variables are expressed as means ± standard deviation (SD), median, range, and the standard error of mean (SEM). Baseline continuous variables were compared between PD and HC using the non-parametric Wilcoxon-Mann-Whitney-Test, as some were not normally distributed. For the binary variables, Fisher’s exact test was used. Longitudinal modeling was done via a random slope/intercept linear mixed model (R-package lme4). The considered follow-up period was six years for PD and four years for HC. ROC curve analysis and area under the curve (AUC) values were calculated (R-package, pROC) and the significance level was set to alpha = 5% for all statistical tests. To evaluate the differences in the biomarker CSF levels of the NTK panel with regard to a positive CSF signature of typical AD core markers, we performed an analysis, building two groups of participants showing normal or changed levels of a-beta and p-tau, using established cut-off values of the AD core biomarkers based on the Elecsys system. For a-beta, the Amsterdam mixed dementia cohort calculated data-driven cut-offs using Gaussian mixed modelling, leading to a cut-off level of 680 pg/mL [17]. For p-tau in CSF, the most established value is 24 pg/mL, which is based on the ADNI study [18].

To assess potential differences in the CSF biomarker levels of cognitively impaired subjects and participants with normal cognitive function, we built two groups based on the cut-off score MoCA <26, indicating a relevant cognitive deficiency.

All analyses were performed with the statistics software R (version 3.6.3; R Core Team 2018).

### Correlation analysis

For the correlation analysis, we calculated the Spearman’s rank coefficient, using a significance level of alpha = 5%. We performed correlation analysis between all the NTK analytes, AD core markers, and also with the clinical markers MDS-UPDRS part III/Total Score and the MoCA score at the last visits.

## Results

### Subject characteristics at baseline

CSF samples from 252 PD patients (mean age 61 ± 9.8 years, 65.5% male) and 115 HCs (mean age 62 ± 11.0, 64.3% male) were analyzed at BL and after 6, 12, 24, 36 and 48 months of follow-up. (Details are shown in S1 Table). Cognitive test scores were slightly lower in PD than HC but still fell within a normal range. In total, 69 PPMI participants developed a relevant cognitive impairment with a MoCA score < 26 during 48 months of follow-up. Detailed BL characteristics are shown in **Table 1**.

**Table 1:** Baseline characteristics of Parkinson`s Disease (PD) patients and Healthy Controls (HC).

Parameter	Level	Parkinson’s Disease (PD)	Healthy Control (HC)	p-value
**N**		252	115	
**Sex**	female	87 (34.5%)	41 (35.7%)	0.93
	male	165 (65.5%)	74 (64.3%)	
**Age (years)**	Mean ± SD	61 ± 9.8	62 ± 11	0.66
	median (min; max)	62 (34; 85)	62 (31; 84)	
**MDS-UPDRS subscore part III**	Mean ± SD	20 ± 8.5	1.4 ± 2.4	< 0.01
	median (min; max)	19 (4; 46)	0 (0; 13)	
**MDS-UPDRS Total Score**	Mean ± SD	32 ± 13	5.2 ± 4.4	< 0.01
	median (min; max)	30 (7; 70)	4 (0; 20)	
**MoCA score**	Mean ± SD	27 ± 2.2	28 ± 1.1	< 0.01
	median (min; max)	27 (17; 30)	28 (27; 30)	
**CSF α-Synuclein (pg/ml)**	Mean ± SD	103 ± 48	127 ± 54	< 0.01
	median (min; max)	95 (14; 371)	122 (31; 335)	
**CSF GFAP (ng/ml)**	Mean ± SD	6.4 ± 3.5	6.9 ± 3.4	0.16
	median (min; max)	5.8 (1.7; 34)	6.4 (1.6; 19)	
**CSF IL-6 (pg/ml)**	Mean ± SD	4.1 ± 3	4.4 ± 4.9	0.48
	median (min; max)	3.3 (1.6; 24)	3.7 (1.5; 49)	
**CSF NfL (pg/ml)**	Mean ± SD	101 ± 56	99 ± 55	0.81
	median (min; max)	87 (26; 451)	90 (9.2; 442)	
**CSF S100 (ng/ml)**	Mean ± SD	1.1 ± 0.33	1.1 ± 0.3	0.21
	median (min; max)	1.1 (0.44; 3)	1.1 (0.46; 1.9)	
**CSF sTREM2 (ng/ml)**	Mean ± SD	6.9 ± 2.2	7.3 ± 2.7	0.27
	median (min; max)	6.6 (2.4; 17)	7 (2.2; 19)	
**CSF YKL40 (ng/ml)**	Mean ± SD	124 ± 47	138 ± 59	0.06
	median (min; max)	118 (31; 320)	125 (40; 336)	

MDS-UPDRS results showed significant differences between PD and HC. Only αSyn showed a significantly lower level in PD than HC. CSF, cerebrospinal fluid; GFAP, glial fibrillary acidic protein; IL-6, interleukin-6; MDS-UPDRS, Movement Disorders Society-Unified Parkinson’s Disease Rating Scale; MoCA, Montreal Cognitive Assessment; NfL, Neurofilament light; SD, standard deviation; sTREM2, soluble triggering receptor expressed on myeloid cells 2; YKL40, chitinase-3-like protein 1.

### Assessment of genetic risk factors

We analyzed the cohort for genetic variants known to increase AD and PD risk or disease severity. For ApoE stratification, we analyzed the available data (142 of 252 PD patients). The cohort showed the following distribution: e2/e2: 2 subjects, e2/e4: 5 subjects, e3/e2: 16 subjects, e4/e3: 35 subjects, e4/e4: 4 subjects. Thus, only four participants had a homozygous status of the most relevant ApoE4 mutation.

None of the patients showed the rare genetic risk variant R47H (rs75932628) known to increase sTREM2 CSF levels. Further, none of the subjects was a mutation carrier of GBA, LRRK2, and SNCA, the most relevant risk genes for PD.

### Biomarker levels

At BL, levels of αSyn in CSF were significantly lower in PD compared to HC (mean: 103 ± 48 pg/ml vs. 127 ± 54 pg/ml, p<0.01) (**Table 1**). The other biomarkers were not significantly different (p>0.05) showing low AUC values of 0.64 for αSyn, 0.57 for YKL40, 0.55 for GFAP, 0.54 for S100, 0.54 for sTREM2, 0.53 for IL-6, and 0.49 for NfL (S1 Fig). The analysis of the differences between the NTK Panel CSF markers between the two groups with normal and positive CSF signature of AD core parameters revealed slight differences e. g., elevated sTREM2 and YKL-40 values in PD patients with “typical” AD core biomarkers against those without. Nevertheless, these differences were not significant. The Box Whisker plots are shown in S2 Fig.

**Fig 1:**
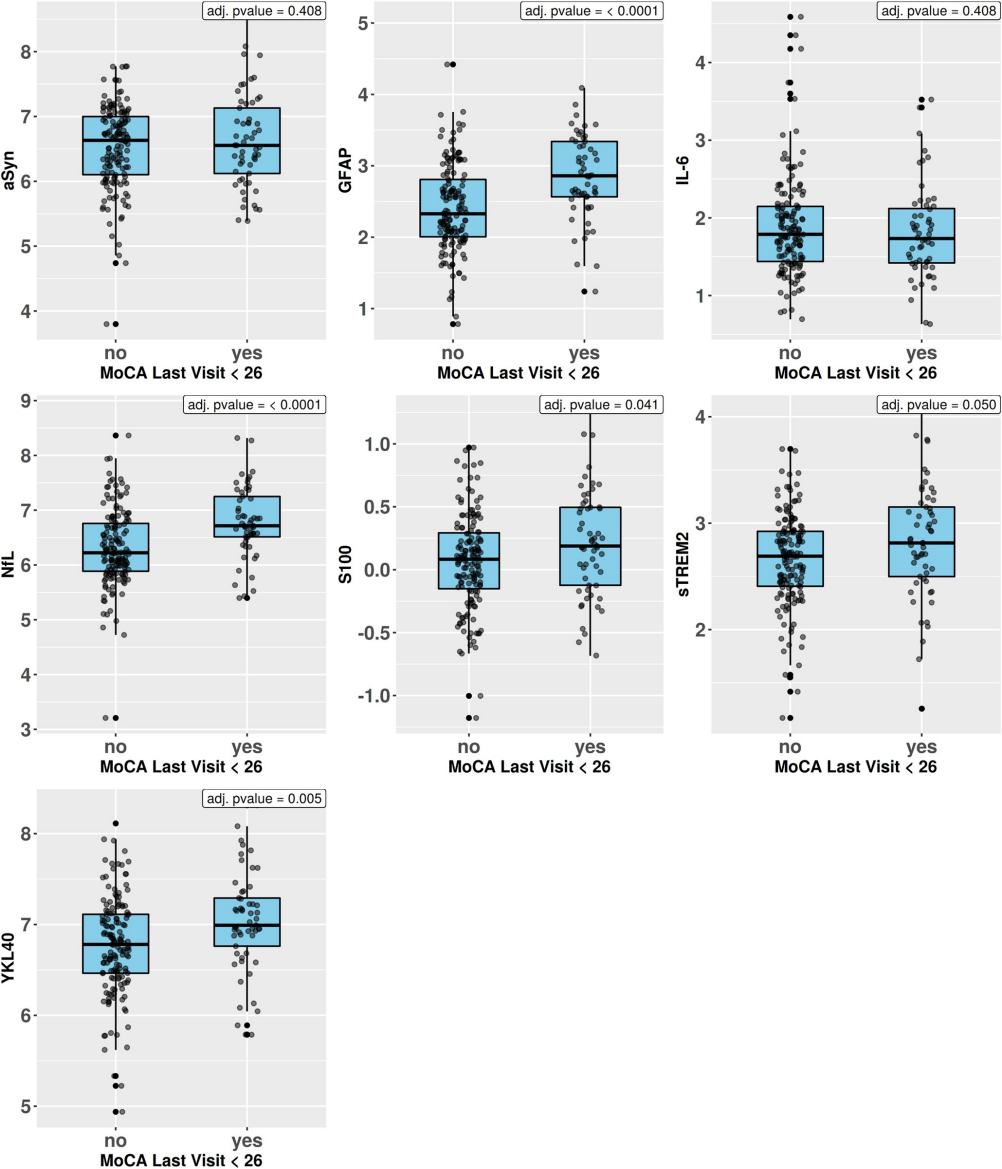
CSF biomarker levels between PD-MCI subjects (MoCA <26) and cognitively unimpaired subjects. αSyn, α-synuclein; GFAP, glial fibrillary acidic protein; IL-6, interleukin-6; NfL, Neurofilament light; sTREM2, soluble triggering receptor expressed on myeloid cells 2; YKL40, chitinase-3-like protein 1.

In the assessment of potential differences in CSF biomarker levels of cognitively impaired participants (MoCA score <26) and those with a MoCA score >26, we found significantly increased CSF biomarker levels for the markers GFAP (p<0,0001), NfL (p<0,0001), S100 (p<0,05), sTREM2 (p<0,05) and YKL40 (p<0,01) in the PD-MCI group. The results are shown in Fig 1.

### Longitudinal modelling

**Fig 2:**
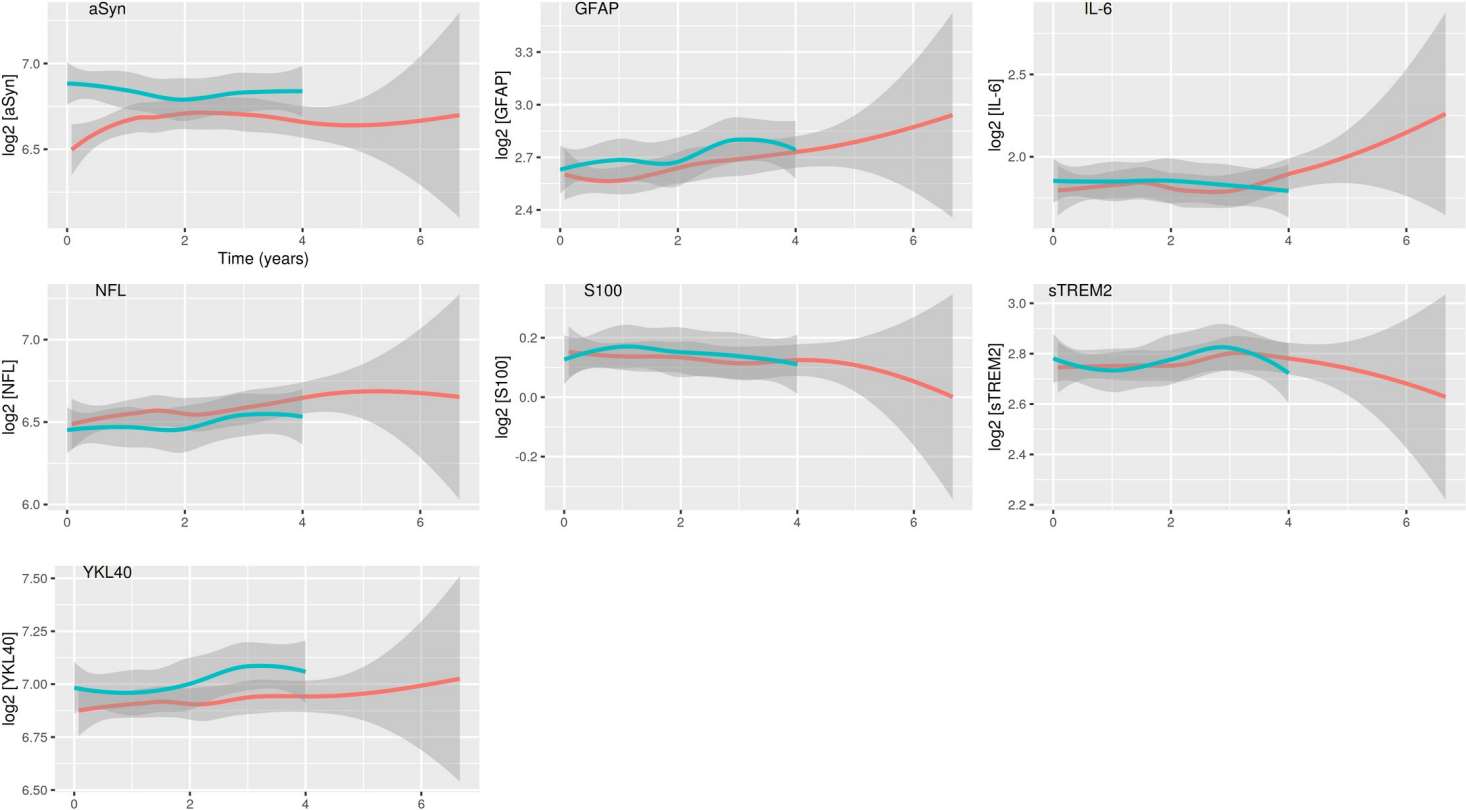
Longitudinal changes of the CSF levels of the tested markers. PD: red, HC: green, The gray ribbon gives estimates of the standard error, the solid line gives a loose fit of the measured data points. The figures illustrate that there is no discrimination in PD versus HC with the NTK panel in the longitudinal profile. Time dependence was calculated with a linear mixed model and found no significant longitudinal alteration of the seven Roche NTK biomarkers. αSyn, α-synuclein; GFAP, glial fibrillary acidic protein; IL-6, interleukin-6; NfL, Neurofilament light; sTREM2, soluble triggering receptor expressed on myeloid cells 2; YKL40, chitinase-3-like protein 1.

Longitudinal modelling showed no significant progression of any of the six biomarker levels. Only the CSF levels of αSyn (p<0.001) were significantly lower at BL in PD and remained so during the follow-up visits; however, they did not show significant longitudinal change versus HCs (**Fig 2**).

Levels of GFAP and IL-6 increased non-significantly in both groups over time. NfL levels did not change over time. S100 and sTREM2 decreased non-significantly during the follow-up visits.

### Correlation analysis

Analysis using Spearman’s R revealed significant positive correlations between all the NTK analytes (except IL-6, S100). The level of PD marker, αSyn, was significantly positively correlated with the levels of IL-6, NfL, YKL40, GFAP, sTREM2, and S100 with coefficients between 0.2 and 0.5. The correlations between the NTK markers and the clinical scores, MDS-UPDRS part III/Total Score, were positive but non-significant. The MoCA score showed a significant negative correlation with αSyn, NfL, and YKL40 (S2 Fig).

**Fig 3:**
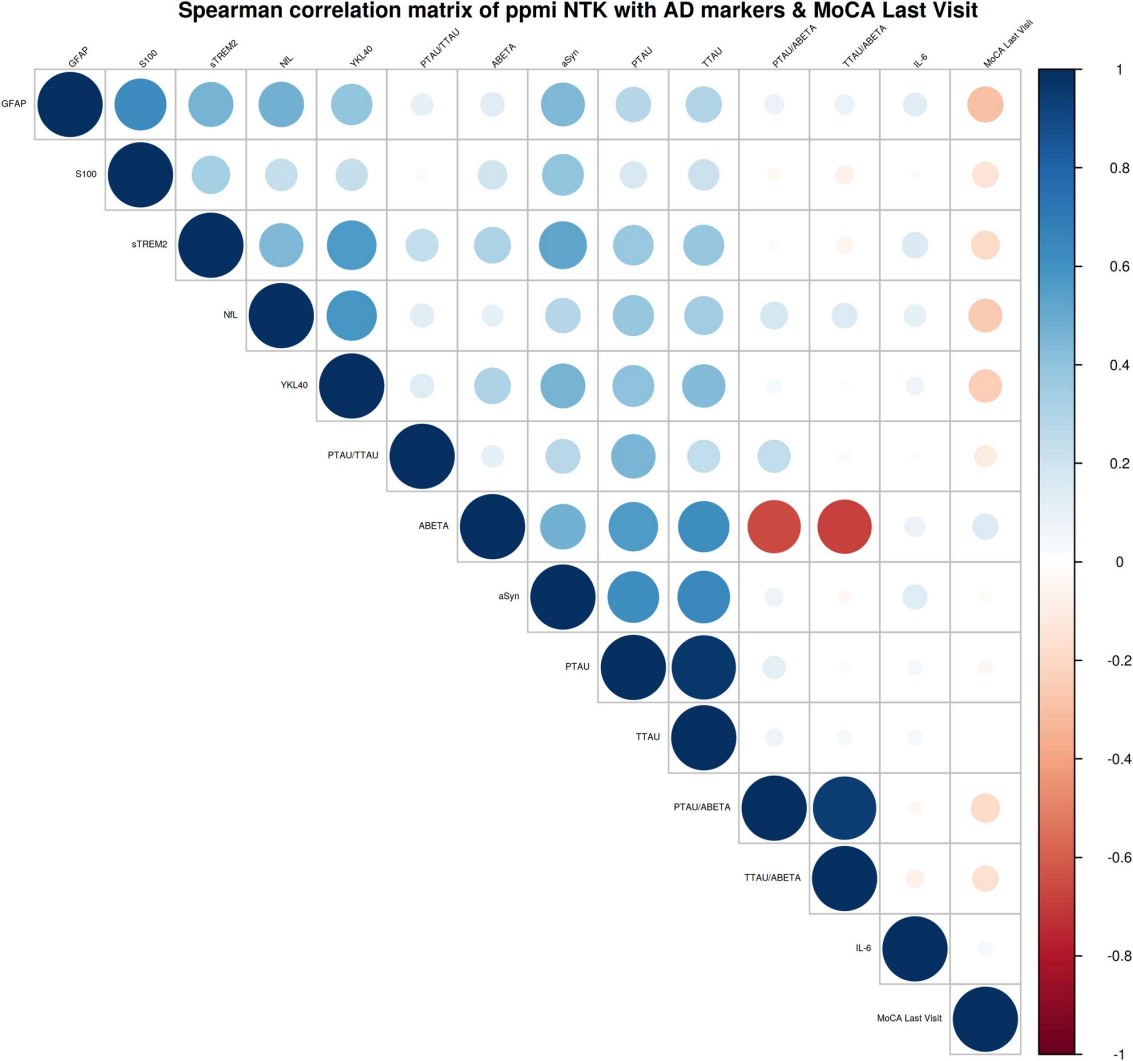
Spearman’s correlation matrix of the NTK biomarkers, the “typical” AD core parameters, and MoCA Score at the last visit. p-tau: phospho-tau t-tau: total-tau, αSyn: α-Synuclein (αSyn), sTREM2: soluble triggering receptor expressed on myeloid cells 2, GFAP: glial fibrillary acidic protein, YKL40: chitinase-3-like protein 1, S100, MoCA last visit, Montreal Cognitive Assessment at last visit.

We performed a correlation analysis including the Montreal cognitive assessment (MoCA) score at the last visits. The results are shown in the Spearman’s correlation matrix below (Fig 3 and S2 Table). It shows significantly negative correlations with the markers aSyn (-0.17, p<0.05), GFAP (-0,31, p<0.0001), NfL (-0.27, p<0.001), S100 (-0.18, p<0.01), sTREM2 (-0.19, p<0.01), YKL40 (-0.22, p<0.01), p-tau (-0.19, p<0.01), p-tau/a-beta (-0.30, p<0.0001) and t-tau/a-beta (-0.26, p<0.001), indicating that impaired cognitive function is associated with higher CSF levels in PD.

The extended analysis, based on Spearman’s rank, correlating the AD core biomarkers p-tau, t-tau and a-beta with the markers of the NTK panel, revealed significantly positive correlations for the following markers: neurofilament light (NfL) (p-tau 0.37 p < 0.0001, t-tau 0.38 p<0.0001, p-tau/a-beta 0,19 p<0.01, t-tau/a-beta 0,19 p<0.01,) α-Synuclein (αSyn) (p-tau 0.66 p = 0.0001, t-tau 0.66 p<0.0001), soluble triggering receptor expressed on myeloid cells 2 (sTREM2) (p-tau 0.46 p = 0.0001, t-tau 0.46 p = 0.0001), glial fibrillary acidic protein (GFAP) (p-tau 0.34 p<0.0001, t-tau 0.37, p<0.0001) chitinase-3-like protein 1 (YKL40) (p-tau 0.52, p<0.0001, t-tau 0.54, p<0.0001, p-tau/a-beta 0.20, p<0.01, t-tau/a-beta 0.18, p<0.01) and S100 (p-tau 0.18, p<0.05, t-tau 0.22, p<0.01). The Spearman’s correlation matrix is shown in Fig 3, the results are shown in S2 Table.

## Discussion

NTK is a previously established biomarker panel that has been validated in a large, longitudinal cohort of 2743 early AD patients, where multiple CSF biomarkers were significantly altered [10, 11]. A decrease of 10–15% in CSF αSyn has been reported previously in PD compared to HCs [19] and was validated here with an independent assay and method. However, this is the first longitudinal CSF study on PD and HC for this biomarker panel. Besides αSyn, there were no significant differences in other analyzed biomarkers.

NTK findings in AD are relevant to discuss because even if they are two separate disease entities, AD co-pathology can often be found in PD and there are common features of neurodegeneration in PD and AD (>50% of AD patients have a Lewy body pathology) [6, 7]. AD and PD are the two most common neurodegenerative disorders based on cognitive decline after an extended preclinical phase of amyloid β (a-beta) and tau proteins aggregates (AD) and motor impairment based on a-synuclein aggregation, forming Lewy bodies (PD). Studies have shown that 78% of PD patients develop cognitive decline on average eight years after disease onset [7]. There are strong findings that support the theory of protein co-aggregation and pathological mechanisms triggering each other. This includes an acceleration of α-syn pathogenesis, and its spreading through the brain induced by a-beta deposits and p-tau induction based on α-syn. This leads to relevant neuronal loss and correlates with cognitive and motor decline [20]. The marker NfL strongly predicted cognitive decline in a-beta-positive individuals, but we did not demonstrate an NfL effect on cognition in PD here. Recently it was shown that levels of NfL in 514 serum samples measured by a different immunoassay differed significantly between PD and HC, increased over 72 months, and significantly correlated positively with clinical motor scores and negatively with cognitive scores [21]. In the same independent longitudinal cohort, CSF NfL levels did not show this significant longitudinal increase, possibly due to the smaller sample size (n = 98 PD, n = 61 HC, n = 17 other neurological diseases). Small sample size and the inclusion of only cognitively unimpaired individuals could also be the reason we did not detect a longitudinal difference here. Further, the antibodies of the NTK NfL assay are likely different to the previously applied digital ELISA [21], although the correlation of both assays was fair (r = 0.606, p = <0.01).

None of the biomarkers analyzed here showed a longitudinal change in PD vs. HC despite biomarkers reflecting pathophysiological pathways relevant to PD, e. g. neuroinflammation. NTK biomarkers showed positive correlations with each other, probably due to their involvement in similar pathways. The sTREM2 receptor in CSF was recently shown to be increased in combination with elevated levels of CSF phospho-tau/total-tau as a possible marker for cognitive decline in PD [22].

The neuroinflammatory biomarker YKL40, involved in synaptic degeneration and glial activation, was recently reported to be increased in AD as well as frontotemporal dementia. Consistent with a previous report [23]. Nevertheless, YKL40 and sTREM2 levels in CSF from PD patients did not differ significantly from HCs in our analysis.

Although AD co-pathology and inflammation have been reported in PD [7, 24], the NTK biomarkers measured here did not show significant diagnostic differences or a longitudinal effect. Maybe neurodegeneration in PD initially involves a much smaller amount of neurons than AD, therefore, synaptic damage and neuroinflammation may not be widespread enough to be reflected in CSF. Nevertheless, given the limited sample size, we cannot firmly conclude that the biomarkers analyzed here have limited value as diagnostic/progression biomarkers in PD since significantly larger populations were examined in the AD studies. Furthermore, participant selection (here randomly) may also be relevant if we consider the overlap of clinical phenotypes and different disease stages, as well as variability added by the multicenter design. Individuals without cognitive impairment were enrolled in PPMI; analyses in patients with cognitive decline during continued longitudinal follow-up may provide further information. Evaluation of NTK in a larger cohort, including more advanced PD, is needed to determine the relevance of these biomarkers for PD.

Genetic variations play a major role in the risk of developing AD or PD. Stratification for the Apolipoprotein E4 (ApoE4) variations revealed an increased risk for late-onset AD, associated with an earlier start and more rapid cognitive progression, but our data showed no signs that this plays a major role here. Further, we checked for the R47H variant of sTREM2l leading to increased AD risk and higher sTREM2 CSF levels [14]. Regarding PD, we analyzed variants in the GBA, LRRK2, and SNCA genes that are well known to increase PD risk, disease onset, and severity [15], but none of these variants were found in our patient group.

By analyzing the CSF signatures of the NTK markers in participants with a MoCA score <26 compared to cognitively unimpaired subjects, we found significantly increased CSF biomarker levels GFAP, NfL, S100, sTREM2, and YKL40 in the PD-MCI group.

Further, when we correlated the NTK biomarkers with the MoCA score, we saw significantly negative correlations with most of the markers, except IL-6 and a-beta. This indicates an association between higher CSF levels of axonal damage and glial response and worse cognitive performance, maybe unfolding a predictive potential here. Further and more comprehensive cognitive tests have now been added and will be available with the ongoing follow-up of the cohort.

Similarly, except for αSyn, the additional biomarkers did not differentiate between PD and HC and none of them showed significant longitudinal differences. Indeed, the NTK markers αSyn, GFAP, NfL, S100, sTREM2, YKL40 as well as p-tau and the combinations p-tau/a-beta and t-tau/a-beta, predicted cognitive decline in PD during follow-up, revealed by correlating the CSF levels with the cognitive measurements. These coherences need to be validated in further studies with a greater number of participants, including genetic analysis to assess its role in disease onset, severity, and pathological burden.

## Supporting information

S1 Table Timepoints of CSF PD sample collections and the assessed markers. P-tau: phospho-tau t-tau: total-tau, αSyn: α-Synuclein (αSyn), sTREM2: soluble triggering receptor expressed on myeloid cells 2, GFAP: glial fibrillary acidic protein, YKL40: chitinase-3-like protein 1, S100.
(DOCX)

S2 Table Results of the Spearman’s correlation of the NTK biomarkers, the “typical” AD core parameters, and MoCA Score at the last visit. P-tau: phospho-tau t-tau: total-tau, αSyn: α-Synuclein (αSyn), sTREM2: soluble triggering receptor expressed on myeloid cells 2, GFAP: glial fibrillary acidic protein, YKL40: chitinase-3-like protein 1, S100, MoCA last visit, Montreal Cognitive Assessment at last visit, *p<0.05 **p<0.01 ***p<0.001****p<0.0001.
(DOCX)

S1 Fig Receiver Operating Curves (ROC)-curves with Area under the Curve (AUC) values for the tested markers. No differentiation between the Parkinson’s patients and healthy controls was possible. αSyn, α-synuclein; GFAP, glial fibrillary acidic protein; IL-6, interleukin-6; NfL, Neurofilament light; sTREM2, soluble triggering receptor expressed on myeloid cells 2; YKL40, chitinase-3-like protein 1.
(TIF)

S2 Fig Box whisker plots showing the difference of the CSF biomarker levels according to the positive CSF signature of the “typical” AD core biomarkers Abeta42 and pTAu181. P-tau: phospho-tau t-tau: total-tau, αSyn: α-Synuclein (αSyn), sTREM2: soluble triggering receptor expressed on myeloid cells 2, GFAP: glial fibrillary acidic protein, YKL40: chitinase-3-like protein 1, S100.
(TIF)
