# Supplementary material for: Biomarkers of neurodegeneration and glial activation validated in Alzheimer’s disease assessed in longitudinal cerebrospinal fluid samples of Parkinson’s disease
Source: PLoS One. 2021 Oct 7;16(10):e0257372. doi: 10.1371/journal.pone.0257372 (PMC8496858; doi:10.1371/journal.pone.0257372)
Supplement: S1 Table — P-tau: phospho-tau t-tau: total-tau, αSyn: α-Synuclein (αSyn), sTREM2: soluble triggering receptor expressed on myeloid cells 2, GFAP: glial fibrillary acidic protein, YKL40: chitinase-3-like protein 1, S100. (DOCX) [file pone.0257372.s001.docx]

| PPMI CSF sample Collections | Timepoints (months) | Number of HC CSF samples analyzed at the time point | Number of PD CSF samples analyzed at the time point | Analyzed markers at the timepoint |
| --- | --- | --- | --- | --- |
|  | Baseline (recently diagnosed PD, drug naïve); MoCa >26 | 101 | 214 | p-tau, t-tau, αSyn, sTREM2, GFAP, YkL40, S100 |
|  | 6 | 97 | 155 | p-tau, t-tau, αSyn, sTREM2, GFAP, YkL40, S100 |
|  | 12 | 95 | 193 | p-tau, t-tau, αSyn, sTREM2, GFAP, YkL40, S100 |
|  | 24 | 85 | 196 | p-tau, t-tau, αSyn, sTREM2, GFAP, YkL40, S100 |
|  | 36 | 68 | 152 | p-tau, t-tau, αSyn, sTREM2, GFAP, YkL40, S100 |
|  | 48 | 72 | 144 | p-tau, t-tau, αSyn, sTREM2, GFAP, YkL40, S100 |

**S1 Table:** Timepoints of CSF PD sample collections and the assessed markers; *p-tau: phospho-tau t-tau: total-tau, αSyn: α-Synuclein (αSyn), sTREM2: soluble triggering receptor expressed on myeloid cells 2, GFAP: glial fibrillary acidic protein, YKL40: chitinase-3-like protein 1, S100*
