# Supplementary material for: Biomarkers of neurodegeneration and glial activation validated in Alzheimer’s disease assessed in longitudinal cerebrospinal fluid samples of Parkinson’s disease
Source: PLoS One. 2021 Oct 7;16(10):e0257372. doi: 10.1371/journal.pone.0257372 (PMC8496858; doi:10.1371/journal.pone.0257372)
Supplement: S2 Table — P-tau: phospho-tau t-tau: total-tau, αSyn: α-Synuclein (αSyn), sTREM2: soluble triggering receptor expressed on myeloid cells 2, GFAP: glial fibrillary acidic protein, YKL40: chitinase-3-like protein 1, S100, MoCA last visit, Montreal Cognitive Assessment at last visit, *p<0.05 **p<0.01 ***p<0.001****p<0.0001. (DOCX) [file pone.0257372.s002.docx]

**S2 Table:** Results of the Spearman’s correlation of the NTK biomarkers, *the “typical” AD core parameters, and MoCA Score at the last visit; p-tau: phospho-tau t-tau: total-tau, αSyn: α-Synuclein (αSyn), sTREM2: soluble triggering receptor expressed on myeloid cells 2, GFAP: glial fibrillary acidic protein, YKL40: chitinase-3-like protein 1, S100, MoCA last visit, Montreal Cognitive Assessment at last visit, *p<0.05 **p<0.01 ***p<0.001****p<0.0001*
