# Supplementary figures and images for: Biomarkers of neurodegeneration and glial activation validated in Alzheimer’s disease assessed in longitudinal cerebrospinal fluid samples of Parkinson’s disease
Source: PLoS One. 2021 Oct 7;16(10):e0257372. doi: 10.1371/journal.pone.0257372 (PMC8496858; doi:10.1371/journal.pone.0257372)

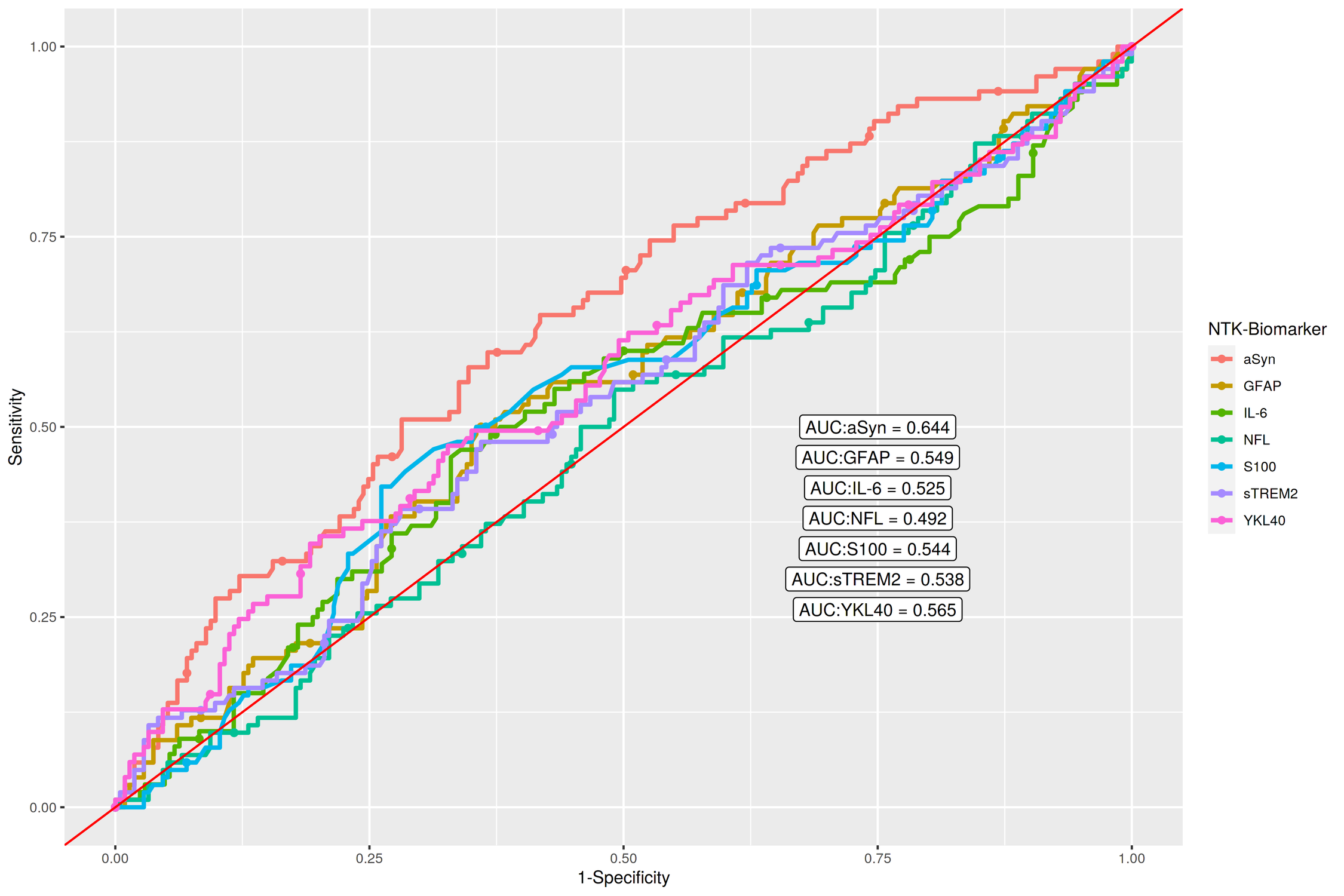

Supplement: S1 Fig — No differentiation between the Parkinson’s patients and healthy controls was possible. αSyn, α-synuclein; GFAP, glial fibrillary acidic protein; IL-6, interleukin-6; NfL, Neurofilament light; sTREM2, soluble triggering receptor expressed on myeloid cells 2; YKL40, chitinase-3-like protein 1. (TIF) [file pone.0257372.s003.tif]

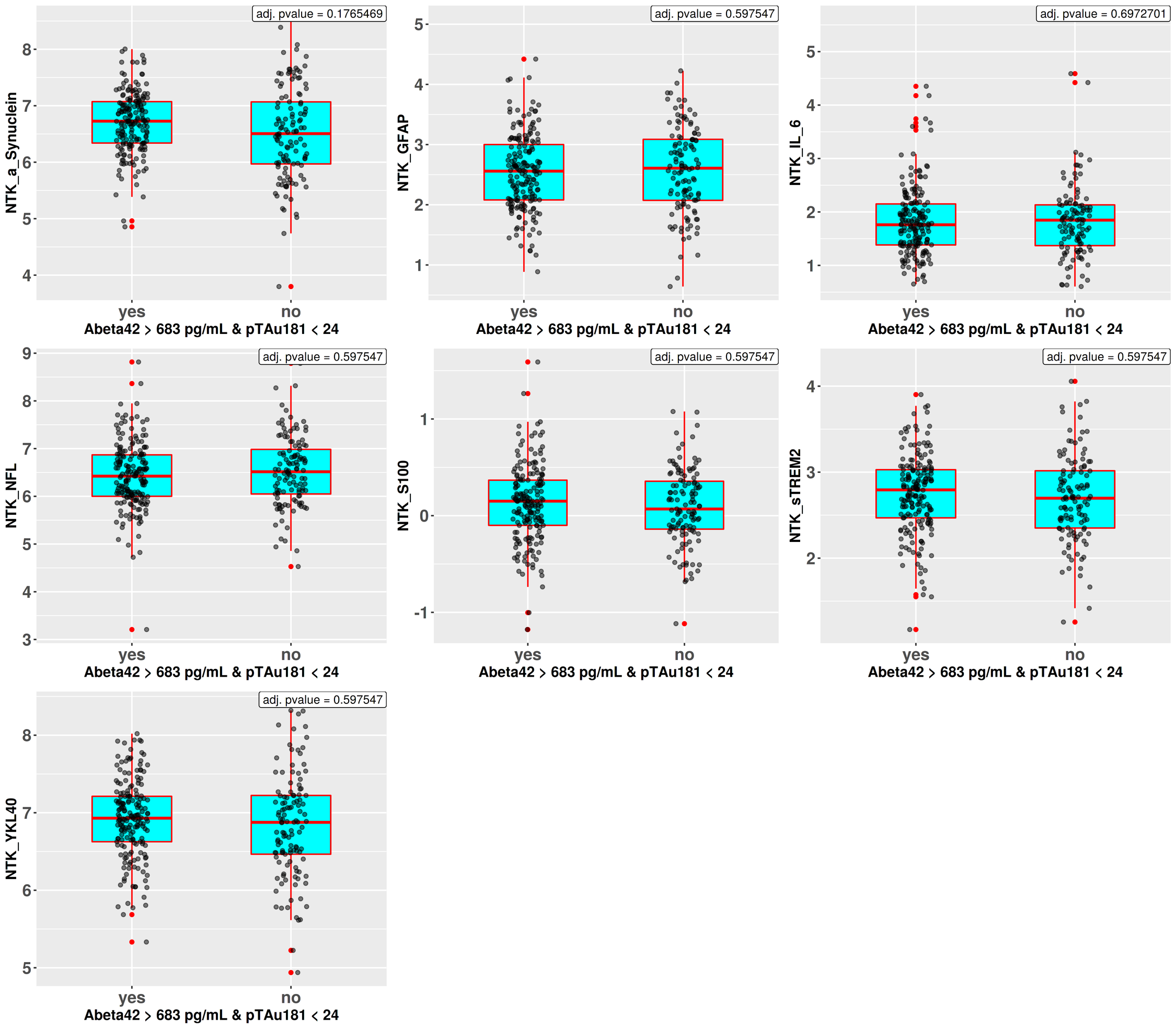

Supplement: S2 Fig — P-tau: phospho-tau t-tau: total-tau, αSyn: α-Synuclein (αSyn), sTREM2: soluble triggering receptor expressed on myeloid cells 2, GFAP: glial fibrillary acidic protein, YKL40: chitinase-3-like protein 1, S100. (TIF) [file pone.0257372.s004.tif]
